# Monkeypox (mpox) in immunosuppressed patients

**DOI:** 10.12688/f1000research.130272.1

**Published:** 2023-02-02

**Authors:** Sirwan Khalid Ahmed, Mona Gamal Mohamed, Eman Abdelaziz Dabou, Israa Abuijlan, Deepak Chandran, Nahed A. El-Shall, Hitesh Chopra, Kuldeep Dhama

**Affiliations:** 1 Department of Pediatrics, Rania Pediatric & Maternity Teaching Hospital, Rania, Sulaymaniyah, Kurdistan Region, 46012, Iraq; 2 RAK College of Nursing, RAK Medical and Health Sciences University, Ras Al Khiamah, United Arab Emirates; 3 Department of Veterinary Sciences and Animal Husbandry, Amrita School of Agricultural Sciences, Amrita Vishwa Vidyapeetham University, Coimbatore, Tamil Nadu, 642109, India; 4 Department of Poultry and Fish Diseases, Faculty of Veterinary Medicine, Alexandria University, Edfina, El-Beheira, 22758, Egypt; 5 Chitkara College of Pharmacy, Chitkara University, Punjab, 140401, India; 6 Division of Pathology, ICAR-Indian Veterinary Research Institute, Bareilly, Uttar Pradesh, 243122, India

**Keywords:** monkeypox, immunocompromised patients, autoimmune disease, human immunodeficiency virus, management, prevention, control

## Abstract

The World Health Assembly declared that smallpox had been completely eradicated from the human population in 1980. Monkeypox, a zoonosis native to damp forested regions in West and Central Africa, is the illness that is most comparable to smallpox clinically and immunologically. Both illnesses could be prevented by the smallpox vaccine. Although the monkeypox virus is a less effective human disease than the smallpox virus, it could now spread among human populations if smallpox had not been eradicated and population-wide immunity had not been developed. A health warning on severe monkeypox in people who are immunocompromised due to Human Immunodeficiency virus (HIV) and other illnesses was released by the U.S. Centers for Disease Control and Prevention (CDC) on September 29, 2022. The advise does not specifically include primary immunodeficiency, but it does define other immunocompromising disorders as “having autoimmune disease with immunodeficiency as a clinical component”. The documented severe signs of monkeypox include widespread rashes with secondary fungal or bacterial skin infections or tissue death (necrosis), intestine obstruction, and difficulties with the heart, lungs, urinary system, and nervous system. Both those with healthy immune systems and those with weakened immune systems, such as those who are immunosuppressed, older people, children, etc., have encountered serious health issues, but the latter group is more likely to do so. According to the advisory, “of the people with severe monkeypox manifestations for whom CDC has been consulted, the majority have had HIV with CD4 counts 200 cells/ml, indicating substantial immunosuppression”. The current article goes into great detail about monkeypox disease occurring in immunocompromised patients and preventive guidelines.

## Introduction

The monkeypox virus (MPXV) is an *Orthopoxvirus* that can spread to humans and result in monkeypox (mpox) disease quite similar to the smallpox. ^1^ Large respiratory droplets, inadvertent or intentional contact with bodily fluids or lesion material or contact with biota—including bedding or towels—are some of the main ways that viruses are spread. Symptoms like malaise, headache, lymphadenopathy, myalgia, and cutaneous symptoms have been documented within one to three days of the onset of fever. Macules may initially be present when a lesion first develops, followed by papules, pustules, and vesicles, which then dry up and peel off. Immunocompromised individuals should be examined for other diseases, such as primary or secondary infection with the varicella zoster virus or other pathogens such as *Cryptococcus neoformans*, *Histoplasma capsulatum*, or *Bartonella henselae*, when skin lesions arise along with viral prodromal symptoms. ^2^ The epidemiological history of the mpox revealed that the first recognition of this disease in human beings was in 1970 in the Democratic Republic of the Congo among children. ^3^ The mpox disease was endemic to central and western Africa region where 12 countries were identified, including Benin, Cameroon, the Central African Republic, the Democratic Republic of the Congo, Gabon, the Ivory Coast, Liberia, Nigeria, Sierra Leone, and South Sudan. ^4^^–^^7^ Since then, human cases of mpox have occurred rarely in that area. ^3^^,^^8^^–^^14^ The first outbreak of the disease reported outside Africa was in the USA in 2003. ^15^ Investigation revealed that the USA outbreak was linked to an infected Prairie dog with MPXV. These pets were housed near rodents shipped from Ghana in 2003. ^16^ After the 2003 USA outbreak, several countries reported mpox cases with a history of travel from Nigeria in 2018, ^17^ including Israel, ^18^ the UK, ^19^ and Singapore. ^20^ Now, some 34 years later, we find ourselves in an eerily similar predicament. ^21^

Since several *Orthopoxvirus* species share genetic and antigenic characteristics, getting infected with one of them may provide significant protection from getting infected with the others. The vaccinia virus vaccine offers defense against illnesses brought on by the variola major, mpox, or cowpox viruses. ^22^^,^^23^ Vaccine-induced cross-protection appears to be mediated by a variety of immunologic pathways, with neutralizing antibodies among the key players. ^24^^–^^26^ Monkeys can be immunized with the human smallpox vaccine to prevent mpox, which is consistent with the ability of the smallpox vaccine to give cross-protection for humans against mpox. Since the end of smallpox vaccinations in 1978, cross-protective immunity to different orthopoxviruses has decreased, especially in younger people without vaccinia-induced immunity, and the number of unvaccinated, susceptible people has increased globally. In fact, during the past few years, these changes have been accompanied by a rise in the number and geographic dispersion of human mpox cases. ^27^^,^^28^ A health warning on severe mpox in people who are immunocompromised due to HIV and other illnesses was released by the U.S. Centers for Disease Control and Prevention (CDC) on 29 ^th^ September 2022. ^29^ Although “having autoimmune disease with immunodeficiency as a clinical component” is included in the advisory's definition of other immunocompromising disorders, primary immunodeficiency is not specifically included in it. There have been reports of widespread rashes, subsequent bacterial or fungal skin infections, tissue death (necrosis), bowel obstruction, heart, lung, urinary, and neurological problems as severe symptoms of mpox. ^25^ Serious problems have happened in both immunocompromised and immunocompetent individuals, although immunocompromised individuals are more prone to experience them. The majority of those with severe mpox presentations for whom the CDC has been contacted have Human Immunodeficieny Virus (HIV) and CD4 counts below 200 cells/ml, indicating significant immunosuppression, according to the advice. ^30^

In-depth discussion of mpox disease in immunocompromised people and recommended preventative measures is provided in the current article.

## The ongoing 2022 mpox outbreak

In the 2022 outbreak, the mpox disease was reported by numerous non-endemic countries. The first case of mpox was reported by the United Kingdom Health Security Agency (UKHSA) in the United Kingdom. Afterwards, cases of the illness were reported in Spain, Portugal, Italy, the United Kingdom, and the United States. ^31^^–^^40^ Up to January 06, 2023, the Centers for Disease Control and Prevention (CDC) confirmed 84,075 mpox cases in 110 countries worldwide with 75 deaths, ^41^ and clinical research has shown that the disease has distinct epidemiological and clinical features. ^31^^,^^32^^,^^42^^,^^43^ Statistics show that one-third of the reported cases were highly concentrated in the USA (the highest), Spain, Germany, and the UK. ^44^ The mortality rate of this disease is less severe as investigation revealed that the MPXV genome involved in the current outbreak belongs to West African clade II, which is less virulent than the Central African clade also known as the Congo Basin clade. ^45^ The combined case fatality rate of the West African and Congo Basin MPXV clades is around 8.7% worldwide. ^46^^,^^47^

The demographic characteristics of reported cases distinguished from the previous outbreak were 99% male with a median age of 37 years, and 44% were HIV-positive. ^48^ Furthermore, 99% of reported cases were gay, bisexual, and having men-to-men sex, ^48^ indicating sexual transmission as a new mode of transmission. ^27^ Interestingly, Pluart *et al*. declared that mpox occurred among healthcare workers. ^49^ However, the protection of healthcare workers shoud be a top prority during this public health emergency. ^50^^,^^51^ Scientists have espoused that the affected people represent the population who did not receive smallpox vaccination due to disease eradication, indicating its possible contribution to their susceptibility to mpox disease. ^52^^,^^53^

Genital lesions have been reported as one of the distinguishing features of the current outbreak. ^54^ The majority of confirmed cases have reported to clinics with at least one symptom, including systemic rashes, fever, or genital rashes, which is not consistent with the typical presentation of mpox disease, which usually includecentrifugal rashes preceded by fever and swollen lymph nodes. ^37^^,^^46^ In addition, the number of rashes is less in most cases, with the first rash occurring in the genital and perineal area followed by monographic and centrifugal distribution all over the body. ^23^^,^^55^ The other sites of lesions were as follows: 55% on the truck, arms, and legs, 25% on the face, 10% on the palms and soles. ^43^ Most rashes (58%) were identified as vesiculopustular. ^43^

## Transmission and replication

The MPXV is considered one of the largest DNA viruses ^56^ and its size ranges from 130 to 360 kbp. ^57^ It therefore stimulates the host immune system rapidly. The MPXV has two distinctive sets of modulatory proteins responsible for invading the immune system, including intracellular modulatory proteins and extracellular modulatory proteins. Another unique feature of MPXV is its exclusive replication in the cytoplasm. ^57^ The replication process of MPXV starts with viral attachment initiated by glycosaminoglycans including chondroitin, heparin sulfates, and laminin, ^58^ followed by viral fusion to the host cell involving 11 to 12 non-glycosylated, transmembrane proteins. ^59^ Once the viral entry is complete, viral transcription takes place by virus-encoded multi-subunit DNA-dependent RNA polymerase. ^60^ It is proceeded by the translation of proteins on the host ribosome, which occurs at three levels: early, intermediate and late translation. ^60^ Studies reveal that DNA synthesis and replication occur at cytoplasmic structures called factories, ^18^ proving its effectiveness in viral RNA synthesis. ^56^ Each factory derives from the cell's rough endoplasmic reticulum (RER). ^61^ Lastly, some of the mature virions transported via microtubules and coated by endoplasmic reticulum or Golgi-derived membranes will exit the cell by fusing with the cytoplasmic membrane and become extracellular enveloped virus (EEV). ^62^

The orthopoxviral physical stability contributes to their varied modes of transmission. ^45^ The mpox information chain includes animal-to-human transmission and human-to-human transmission. Since the virus is present in the infected host lesions, crust, and secretions, direct contact with these secretions can promote viral transmission, whether the host is animal or human. Animal to human transmission occurs when there is direct contact with the bodily fluids of affected animals, such as blood, saliva, and cutaneous and mucosal lesions of these animals. Furthermore, respiratory droplets and eating raw meat or poorly cooked meat products of affected animals can transmit the MPXV. Another transmission mode includes the bites and scratches caused by infected animals. ^63^ Monkeys, rats, and squirrels are the primary host of MPXV in Africa. ^51^

Similarly, transmission from human to human occurs when there is direct contact with an infected person's rashes, sores, scabs, respiratory droplets, or oral fluids. ^44^^,^^46^ Furthermore, sharing the same household of infected individuals increases the risk of contamination. ^63^ The common mechanism used by MPXV in the 2022 outbreak is attacking the host defense by encoded proteins produced during transcriptions named MHC class II antigen presentation inhibitor, an IFN-alpha/beta receptor glycoprotein and IL-1/TLR signaling inhibitor. ^64^ Moreover, vertical transmission, such as from mother to fetus, was also identified. ^65^ Another unique feature that contributed to the current outbreak is the excessive genome mutations that result from the action of apolipoprotein B mRNA-editing catalytic polypeptide-like 3(APOBEC3) enzymes. ^64^ The action of these enzymes results in hypermutated, viable variants of the virus as revealed by phylogenomic analysis. ^64^

In the current outbreak, there is a disappearance of an epidemiological link to the endemic region. ^27^ Yet, data showed that 91% of confirmed cases reported an immediate sexual exposure before symptoms started, particularly with men having sex with men, ^23^ while 98% of the patients identified as gay or bisexual. ^43^ Therefore, WHO considered sexual exposure in the 21 days before symptoms onset as a risk factor, ^44^^,^^46^ especially among men who have sex with other men. Evidence showed that all confirmed cases of mpox among men who have sex with other men have a history of sexually transmitted disease (STD), hepatitis C, syphilis, and HIV. ^33^ Epidemiologists reported that the reproductive ratio value of MPXV is between 1.10 and 2.40, suggesting the ability of an infected individual to infect one to two other persons. ^66^ Such value highlights the need to initiate preventive measures to contain the spread of the disease. ^50^^,^^63^^,^^67^

## Immunosuppression

The immune system plays a vital role in defending the body against microorganisms and other foreign bodies. ^68^ Failure of the immune system to protect the body indicates the body's immunosuppressed status. Immunosuppression is defined as “a state of temporary or permanent dysfunction of the immune response resulting from insults to the immune system and leading to increased susceptibility to disease”, originally proposed by Dohms and Saif in 1984. ^69^^,^^70^ Immune dysfunction is classified as primary and secondary immune dysfunction. The immunosuppression status occurs due to disease condition or medication-induced immunosuppressed state. Consequently, hematologic malignancies, solid-organ transplant, chimeric antigen receptor (CAR)-T-cell therapy, or hematopoietic stem cell transplant are all common causes of immunosuppression, as are moderate to severe levels of immunodeficiency disease, such as DiGeorge syndrome, Wiskott-Aldrich syndrome, HIV cases with CD4 cell counts less than 200/mm ^3^, and the history of an AIDS-defining illness without immune recrudescence. The degree of immunosuppression can vary among patients. ^71^ Furthermore, medication that induces immunosuppression status includes high-dose corticosteroids (i.e., ≥20 mg of prednisone for two or more weeks), alkylating agents, antimetabolites, transplant-related immunosuppressive drugs, tumor necrosis factor (TNF) blockers, and other biologic agents that are immunosuppressive or immunomodulatory. Evidence has shown a steady rise in the number of acquired immunity deficiency (secondary immunodeficiency) conditions in response to an increasing number of individuals with transplantation of solid organ or hematopoietic stem cells ^72^ as such medical treatment requires an intensive immunosuppressive regimen which can cause the patient to develop severe adenovirus infections. ^72^ A US cross-sectional study revealed that 2.8% of patients experienced drug-induced immunosuppression between 2018 and 2019, particularly those who were prescribed oral corticosteroids for 30 days or longer (40.9%). ^73^ Oral corticosteroids, methotrexate, and other disease-modifying anti-rheumatic therapies, transplant antirejection drugs, tumor necrosis factor inhibitors, antineoplastic treatments, and other biological product medications all contribute to immunosuppression. ^73^ Immunosuppressive medication has a significant role in patient survival such as keeping a person from rejecting an organ transplant and treating the overactivity of the immune system in cases like autoimmune disease and allergies. On the other hand, immunosuppressant medication can negatively impact a patients' health as it increases their likelihood of having infections. ^74^

The most commonly reported infection among immunocompromised patients is a protracted infection characterized by the intra-host viral revolution and the generation of multiply mutated viruses. ^74^ The common form of protracted infection is protracted bacterial bronchitis which occurs among children. ^75^ In addition, other types of infections associated with immunosuppression are viral, bacterial, and fungal infections of the blood, lungs, and central nervous system. ^76^ Furthermore, the immunocompromised status might increase patients' susceptibility to prolonged infection, resulting in a prolonged length of stay in hospital and more complications. ^77^ Studies on immunosuppressed patients' response to infection vary in terms of mortality rate, complication, and length of stay. For example, authors of a study conducted in Spain among immunocompromised patients admitted with Influenza A (H1N1) virus in 2009 reported that immunocompromised patients had higher mortality than non-immunosuppressed individuals. ^78^ In addition, the complicated cases experienced a bacterial coinfection, specifically gram-negative bacilli and *Staphylococcus aureus* infections. ^78^ Inversely, a retrospective cohort study in the U.S. in 2020 was conducted on the impact of COVID-19 on drug-induced immunosuppressed patients. It revealed that the chronic use of immunosuppressive drugs was neither associated with worse nor better clinical outcomes among hospitalized cases with COVID-19 in terms of the risk of using mechanical ventilation, in-hospital mortality, or length of stay. ^79^ Similarly, another study revealed that immunosuppressed patients infected with COVID-19 are not at increased risk of severe pulmonary disease compared to other populations, highlighting the necessity of continuing patients' treatment, such as chemotherapy and radiotherapy. ^80^ The controversial condition of immune suppression was pregnancy. There was a debate regarding pregnancy being considered an immunosuppression state. ^81^ However, the authors highlight that the susceptibility of pregnant women to infection depends on the placental immune response to certain pathogens. ^81^^,^^82^

## Monkeypox in immunosuppressed patients

According to studies, there is a greater chance of severe manifestation of MPXV infection in immunosuppressed patients. ^42^^,^^83^^,^^92^^,^^84^^–^^91^ Immunosuppressed patients include patients who have undertaken cancer treatment, organ transplant, HIV infection, primary immune deficiency disorders, some severe autoimmune disorders, and medications to treat autoimmune diseases and other illnesses that can weaken the immune system. The disease appears to have a lethal prognosis, especially in children who have not been vaccinated against smallpox. ^83^ Individuals with compromised immune systems include those with HIV, hematological malignancies, usage of immunosuppressive medicines such corticosteroids, organ transplant patients, autoimmune illness, or innate immunodeficiencies.

Patients with immune impairment run the risk of contracting serious illness. As a result, efforts should be undertaken to strengthen their immune system in addition to using tecovirimat (e.g., limiting the use of immunosuppressive medications, initiating antiretroviral therapy for those with HIV). Following a tecovirimat 14-day therapy, certain individuals with significant immunocompromise may continue to develop lesions. ^93^ If viral resistance is suspected in this situation, physicians may think about sending a second swab for sequencing. As long as there is no sign of viral resistance, it is reasonable to continue tecovirimat (with emphasis on the need for a fatty meal for optimal absorption) until there is clinical improvement, but no longer than 90 days. ^94^ In contrast, if there is sign of viral resistance, a second anti-viral medication, such as cidofovir (or brincidofovir, if it becomes available), can be added. An infectious disease expert or public health official should be consulted for the management of such patients (e.g., the CDC mpox consultation team in the United States. ^26^^,^^29^^,^^67^^,^^95^

The immunocompromised patient is more liable to get the mpox infection with severe manifestations of mpox, including extensive skin rashes with secondary bacterial or fungal infections or necrosis, bowel blockage, and heart, lung, urinary, and neurological complications. ^34^^,^^53^^,^^86^ Guarner *et al*., (2022) ^27^ reported that an immunosuppressed patient had several scattered maculopapular rashes and pustules on the trunk, upper and lower extremities, groin, and peri-anal area and palpable cervical lymph nodes. Moreover, immunocompromised patients are more likely to develop complications, including respiratory deterioration, acute kidney injury, and multiple organ dysfunctions. ^83^ Simon-Gozalbo *et al*. (2022) ^96^ reported a case of a 30-year-old male patient diagnosed with HIV infection. The manifestations were maculopapular rash affecting mainly the trunk, buttocks, upper and lower extremities, and multiple demarcated purpuric macules with central umbilicated pustules and crusts, along with palpable cervical lymph nodes.

## Management and prevention

Prevention is essential for the immunocompromised patient to protect them from infection. There are two types of vaccines: second and third generation. Second-generation vaccines called replication-competent vaccinia virus vaccines due to their replication capacity in mammalian cells (examples include ACAM2000 and APSV approved for >18 years old) are associated with complications among immunocompromised patients. ^97^ This type of vaccine is not recommended for any group of immunosuppressed patients, especially those with inflammatory/autoimmune diseases of the central nervous system where there can be an outbreak of the disease. Third-generation vaccines, for example, MVA-BN/JYNNEOS/Imvamune, are highly attenuated live vaccines. In contrast to replication-competent smallpox vaccines, these replication-deficient vaccinia vaccines pose a lower risk of side effects, making them suitable for use in healthy people as well as those with immune system deficits, HIV infection, atopic dermatitis, or allergic rhinitis. However, those with compromised immune systems may not respond as well to the vaccine. ^98^^,^^99^ The vaccination of those with immunosuppression, who are at high risk for developing mpox sequelae, should proceed with a 0.5 ml SC/IM dose. People living with HIV whose CD4 count is greater than 200 cells/mm ^3^ can get MVA-BN through intradermal administration. ^100^

JYNNEOS (also known as Imvamune and Imvanex) is a vaccine against a modified Vaccinia Ankara virus that does not replicate. In the US, it was approved for use in preventing both mpox and smallpox. ^101^ Because JYNNEOS does not cause the development of live viruses in vaccinated patients, it is safer for use in immunocompromised individuals than ACAM2000. Importantly, however, immunocompromised persons may have a lower immunological response to the JYNNEOS vaccination. Thus, protection can be weaker than in immunocompetent people. Both vaccines can legally be administered to those aged 18 and up. However, information on JYNNEOS's ability to protect humans from MPXV infection is scant. ^26^^,^^46^^,^^102^ There is a lack of information regarding the efficacy of vaccinia immune globulin (VIG) in the treatment of mpox sequelae. In severe cases of human mpox, VIG may be considered, albeit its efficacy is uncertain. In the case of an exposed person with significantly compromised cellular immunity, for whom smallpox vaccination is not an option, VIG may be considered as a prophylactic measure. ^1^

In conclusion, the available vaccines for smallpox today are also approved for mpox due to the 85% effectiveness provided by cross-immunity. However, before recommending or not vaccinating immunosuppressed patients, it is essential to be well informed of the type of vaccine that local authorities are obtaining. If it is a second-generation live attenuated vaccine, these patients should not receive it. If it is a third generation live attenuated vaccine with no replication capacity and proven safe in immunosuppressed patients. In that case, vaccination should be advised, always considering that the underlying disease should not be active at the moment of the inoculation. ^2^^,^^103^

No licensed treatment or proper evidence-based guideline is available for treating human mpox. However, the viruses belonging to the *Orthopoxvirus* genus are genetically similar. Hence, antivirals used to treat smallpox might be effective against mpox. Antiviral drugs such as tecovirimat, cidofovir, and brincidofovir can be considered mainly for those with severe symptoms or who may be at risk of poor outcomes, such as those with immune suppression. ^104^

**Figure 1.**
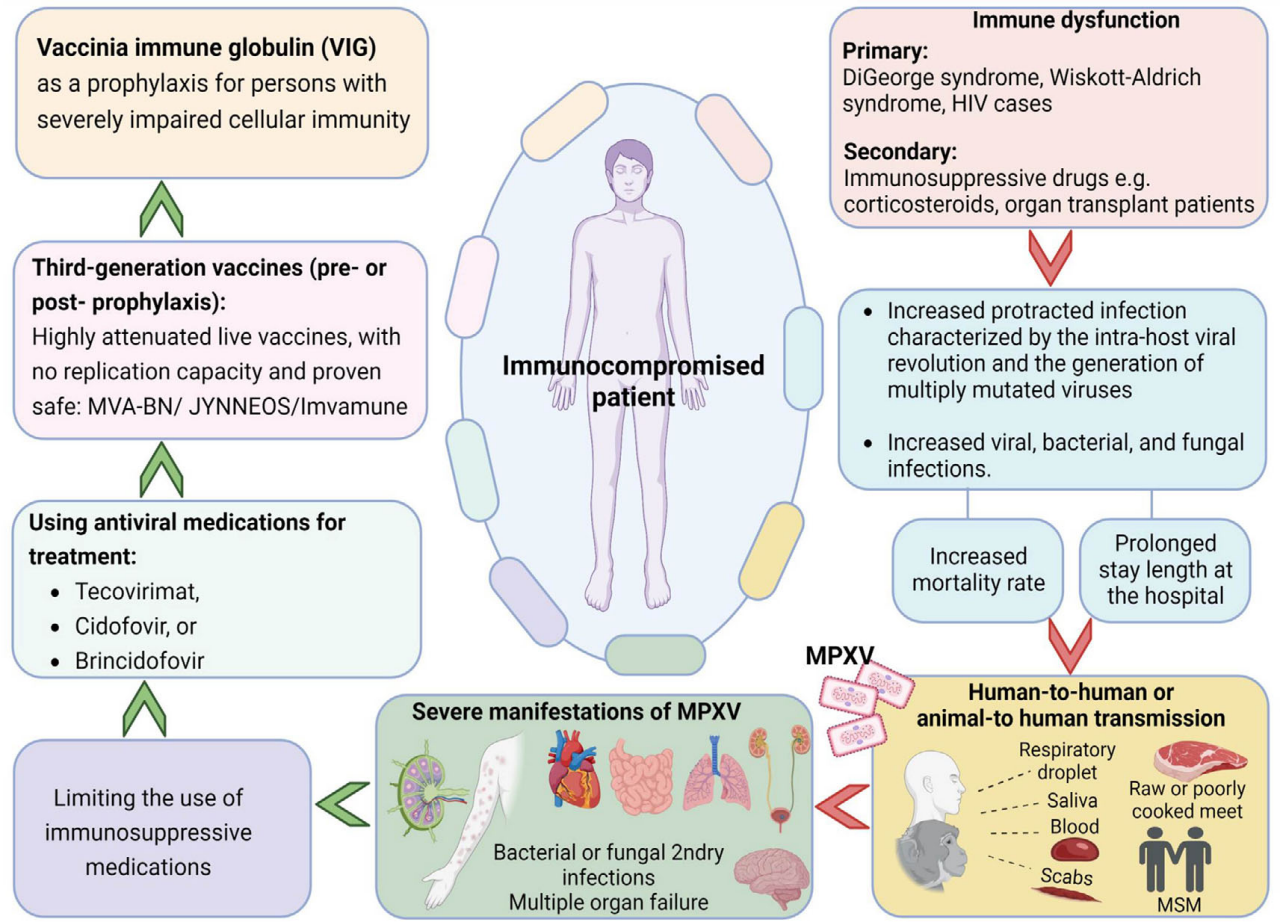
An overview on monkeypox in immunosuppressed patients, prevention and clinical management (Designed with Biorender premium software; https://app.biorender.com/).

Data on mpox in HIV patients are sparse; however, early identification, treatment, and prevention may lessen the severity of potential complications and slow the disease's transmission. ^84^ Two anti-viral medications, tecovirimat and brincidofovir, have been given the green light by the Food and Drug Administration (FDA) to treat smallpox. The Centers for Disease Control and Prevention (CDC) offers tecovirimat for use in the treatment of mumps as a first-line agent. Co-administration of tecovirimat and antiretroviral therapy (ART) for HIV infection is safe because there are no known medication interactions that would prevent it. The JYNNEOS vaccination can be used for prophylaxis both before and after exposure if necessary. Although the European Medicines Agency (EMA) has given its approval for the use of tecovirimat in mpox, there is a need for innovative therapies with different mechanisms due to the danger of resistance. In terms of effectiveness and safety, tecovirimat is the drug of choice for treating mpox. Consequently, the accessibility and diversity of effective anti- *Orthopoxvirus* medicines will be improved through the target-based design of novel antivirals. Patients who did not show a response to tecovirimat run the risk of developing resistance. An immunocompromised HIV/AIDS patient was reported to have contracted mpox by Viguier *et al.* (2022). ^88^ He had a serious, prolonged infection that was treated with tecovirimat for 14 days after his clinical status deteriorated. Almost immediately, he began to feel better, and both the skin lesions and MPXV burdens dropped with no negative consequences. In this instance, tecovirimat shows promise as an effective treatment. The success of tecovirimat in this patient suggests that it may be used to treat other immunocompromised people who have contracted MPXV. Hernandez *et al*. (2022) ^86^ reported a case study about an immunocompromised patient and treated him with tecovirimat. Tecovirimat could effectively and safely treat severe mpox infections among immunocompromised patients. Last but not least, there is an immediate need to focus on minimizing psychological distress, particularly among immunocompromised individuals and healthcare workers. ^95^^,^^105^^,^^106^ An overview of mpox in immunosuppressed patients, preventive measures and clinical management is presented in Figure 1.

## Conclusion and future prospects

Immunotherapeutics and preventative strategies are critical public health interventions that complement rigorous contact tracing in stopping the spread of mpox. In a similar manner, serology-based investigations are effective surveillance tools for tracing contacts and determining exposure histories. However, given that vaccination provides a baseline of protection against poxviruses, these serological diagnostic approaches must be MPXV-specific. Expanding the surveillance network and identifying gaps are also crucial for an efficient ring-fencing system. Importantly, there is a fundamental need in public health to alert individuals who may be exposed about the advantages and hazards of vaccination.

Numerous important scientific queries are yet unanswered. The mechanisms of immune defense against the MPXV will need to be better understood, which will necessitate more research on the human systemic and mucosal immune responses during MPXV infection. It is significant to note that it is not yet known whether previous exposure to variola, mpox, or smallpox vaccine results in any type of mucosal immunity. Understanding the mucosal immune responses will be critical due to the respiratory aerosol transmission of MPXV and other poxviruses. To further understand the respiratory difficulties produced by MPXV, it would be especially necessary to understand the functions of tissue-resident memory T cells and IgA in infection. Because MPXV DNA has been detected in semen, it is also necessary to define the immunity of the preputial mucosa. A key objective for assessing more recent vaccines, particularly those intended for the at-risk populations of immune compromised populations such as older people, pregnant women and children, is defining the immunological correlates of protection. What other factors, such as those related to behavior, geography, nutrition, health, immunology, or genetics, besides not having received a vaccine, could be at play? Recent research suggests that the severity of a viral respiratory infection in young children is tied to the efficiency of their innate immune responses. Children who are infected typically have weaker T cell and B cell responses than adults, similar to reports for SARS-CoV-2 infections. Children with MPXV tend to manifest a more severe disease and have lower vaccine effectiveness, which may be explained by the characterization of adaptive immune responses in these children. Understanding the risks of vaccination in immunocompromised populations, particularly children and expectant women, is also crucial.

It is feasible for MPXV to co-infect with any of the several infectious diseases that are endemic at global level. A co-infection with malaria and an alphavirus, for instance, can drastically alter host immunity and influence the course of an illness. Co-infection with other illnesses that disproportionately affect the group of men who have sex with men makes it especially important to understand mpox disease and immunization, especially given the continuous transmission among these communities in non-endemic countries. This is especially true for HIV-1, which can significantly inhibit adaptive immune reactions. Additionally, it is important to identify the risk factors for severe MPXV. The most vulnerable groups are known to be unvaccinated people, pregnant women, and young children. Other immunocompromised populations, such as older people, those using long-term drugs, and those with underlying metabolic illnesses that may exhibit the disease in a different way, also need to be characterized though.

Patients, including immunocompromised humans such as geriatric patients or babies born to mothers infected with (MPXV) should be closely monitored for the development of chronic health problems. The Zika virus epidemic provided evidence that children who were exposed to the virus in utero may be at risk of developmental issues later in life. ^107^ Evidence suggests that just 25% of pregnant women infected with MPXV would be successful in giving birth ^108^; there should be a higher priority placed on this area of research. Furthermore, infants and young children appear to be more vulnerable to severe mpox. ^109^^,^^110^ Data on fetal development after congenital MPXV infection are currently missing. The ability to determine whether MPXV infection can have long-term repercussions, as was seen after SARS-CoV-2 infection during the current pandemic, would also be made possible by longitudinal surveillance of patients with the MPXV.

## Data Availability

No data are associated with this article.
