# Supplementary material for: Monkeypox (mpox) in immunosuppressed patients
Source: F1000Res. 2023 Apr 3;12:127. Originally published 2023 Feb 2. [Version 2] doi: 10.12688/f1000research.130272.2 (PMC10113800; doi:10.12688/f1000research.130272.2)
Supplement: Peer review history

# Peer review: Reviewer response for version 2

**DOI:** 10.5256/f1000research.146045.r168601

**Published:** 2023-04-26

**Authors:** Jawad Basit

**Affiliations:** 1 Department of Medicine, Rawalpindi Medical University, Rawalpindi, Pakistan

The recent revisions by the authors have added quality to the paper. The authors have incorporated all the feedback in the revised manuscript. This article would be a clinically relevant addition to the existing literature on the given topic. My final verdict is 'Accept'. No minor or major revisions are required.

Is the review written in accessible language?

Yes

Are all factual statements correct and adequately supported by citations?

Yes

Are the conclusions drawn appropriate in the context of the current research literature?

Yes

Is the topic of the review discussed comprehensively in the context of the current literature?

Yes

Reviewer Expertise:

Cardiology, Novel infections, Malignancies and hematology, gastroenterology

I confirm that I have read this submission and believe that I have an appropriate level of expertise to confirm that it is of an acceptable scientific standard.

# Peer review: Reviewer response for version 2

**DOI:** 10.5256/f1000research.146045.r168603

**Published:** 2023-04-18

**Authors:** Dattatreya Mukherjee

**Affiliations:** 1 International School, Jinan University, Guangzhou, China

Authors have updated the manuscript as per my review comments. 

 All my review comments are addressed. Thanks to all the authors for updating the manuscript as per my review comments. Also, thanks to the editor for selecting me as a reviewer.

Is the review written in accessible language?

Yes

Are all factual statements correct and adequately supported by citations?

Yes

Are the conclusions drawn appropriate in the context of the current research literature?

Yes

Is the topic of the review discussed comprehensively in the context of the current literature?

Yes

Reviewer Expertise:

Public Health, Infectious Disease

I confirm that I have read this submission and believe that I have an appropriate level of expertise to confirm that it is of an acceptable scientific standard.

# Peer review: Reviewer response for version 2

**DOI:** 10.5256/f1000research.146045.r168602

**Published:** 2023-04-13

**Authors:** Osaretin Okonji

**Affiliations:** 1 School of Pharmacy, University of the Western Cape, Cape Town, South Africa

The authors' revision have improved the manuscript. The update and the additional information makes the review article more relevant. There are still minor edits issues in the manuscript, so I recommend that the authors closely evaluate for these. I suggest proper editing of the manuscript. Below are few issues and editing issues. 

 Third paragraph under transmission and replication: ‘The source of MPXV has not been determined, however rats are a leading possibility’

- The authors’ should revised for clarity, stating that the evidence of mpox virus infection  has been observed in animals including rats, squirrels, monkeys…………….

Prevention strategy section

- Paragraph 5, please revised ‘up’ for clarity- those aged 18 and up. 

Immunosuppressed patients

- Second paragraph: Please put the reference - A CDC investigation of 57 people…………

"Sexual contact was suspected as the mode of transmission in 95% of the 528 instances of mpox reported, with 98% of patients being gay or bisexual men. There were no fatalities, although 13% of patients needed hospitalization due to discomfort; a few patients needed hospitalization due to visual lesions, decreased oral intake, myocarditis, or acute kidney disease. The majority of patients exhibited a rash upon presentation, with the majority having less than 10 lesions and 11% having more extensive lesions. Patients had a 41% HIV infection rate, but after receiving antiretroviral medication, their viral loads dropped to undetectable levels. Most importantly, no coexisting immunocompromising diseases were observed, indicating that this is an immunologically healthy group."

- Please include a reference, and indicate where this result came from, is it a global report or CDC, or WHO... for clarity.

"bedding or towels—are some of the main ways that viruses are spread."

- lease be more specific here 'that viruses' do you mean mpox virus

Third paragraph under. Transmission: "Patients can be exposed to you through indirect contact if you are in,,,"

- Please be specific for clarity

"Repetition of consumption of raw meat, body fluids......" in the second and third paragraph in the transmission and replication.

- Succinctly join the 2 paragraph as 1. 

"A CDC investigation of 57 people hospitalized with severe mpox showed a substantially more dismal natural history among immunocompromised persons. Around 90% of people had CD4 counts 200 cells/mm3, and roughly 70% had CD4 levels 50 cells/mm3. Less than 10% of people were getting antiretroviral therapy. Just about 5% of patients had undergone a solid organ transplant, and another 3.5% had been diagnosed with a hematologic malignancy. Severe mucosal lesions were present in the majority of patients, and all of them had severe skin lesions. In addition, mpox often spread to other organs, affecting the lungs (21%), eyes (7%) and brain (7%). Patients were treated with tecovirimat (93%), vaccinia immune globulin intravenous (51%), or cidofovir (23%), all of which are antivirals that target mpox. "

- This please write succinctly only important details relevant to this report and add reference.

Is the review written in accessible language?

Yes

Are all factual statements correct and adequately supported by citations?

Yes

Are the conclusions drawn appropriate in the context of the current research literature?

Partly

Is the topic of the review discussed comprehensively in the context of the current literature?

Yes

Reviewer Expertise:

NA

I confirm that I have read this submission and believe that I have an appropriate level of expertise to confirm that it is of an acceptable scientific standard, however I have significant reservations, as outlined above.

# Peer review: Reviewer response for version 1

**DOI:** 10.5256/f1000research.143016.r162331

**Published:** 2023-02-21

**Authors:** Dattatreya Mukherjee

**Affiliations:** 1 International School, Jinan University, Guangzhou, China

Thank You for Giving me the opportunity for reviewing the manuscript. This is a very nicely described article. My comments are given below:

 1. Introduction is written in detail. In the introduction please add about the MPOX in immunocompromised patient (in short). If there is any published cases on it, then authors can write it in short in the introduction part. 

 2. "The first case of Mpox was reported by the United Kingdom Health Security Agency (UKHSA) in the United Kingdom." Please add a reference. 

 3. An image of mechanism can be added at Transmission and replication part. (Pathophysiology).

 4. In Ongoing MPXV outbreak 2022 part, one image of world map can be added with the color differentiation of cases. 

 5. In HIV, can we see CD4 count before injecting the vaccine? In that case, in what CD4 count, we can give vaccine? Is there any study on it? If not this paper can recommend this idea for the future. (If authors support my concept of CD4).

 6. Conclusion and future aspects are written very well. 

 7. The paper has a good discussion on Mpox Vaccine. If authors agree my idea about CD4, can add as a recommendations

 8. Is it possible to have an info graph on the topic? The Clinicians will get to know the treatment procedure quickly in ward. Like if an immunocompromised patient got admitted with Mpox. How the treatment should be given. What are the drug of choices. PCR is the test of Choice, please add that also in the management and info graph parts. 

 This is a nicely written and detail review on this topic. Information are written nicely with proper citations. This is a much needed discussion and the paper has filled that void successfully. This paper will be very much helpful for the scientific societies.

Is the review written in accessible language?

Yes

Are all factual statements correct and adequately supported by citations?

Yes

Are the conclusions drawn appropriate in the context of the current research literature?

Yes

Is the topic of the review discussed comprehensively in the context of the current literature?

Yes

Reviewer Expertise:

Public Health, Infectious Disease

I confirm that I have read this submission and believe that I have an appropriate level of expertise to confirm that it is of an acceptable scientific standard, however I have significant reservations, as outlined above.

# Response

**Published:** 2023-03-23

**Authors:** Sirwan Ahmed

**Affiliations:** Rania Pediatric & Maternity Teaching Hospital, Iraq

This is a nicely written and detail review on this topic. Information are written nicely with proper citations. This is a much-needed discussion and the paper has filled that void successfully. This paper will be very much helpful for the scientific societies.

 Answer: Authors are very thankful to the reviewer for devoting time for improving the quality of the manuscript. The inputs and suggestions from the reviewer have been taken under consideration and have been implemented in the manuscript.

 1. Introduction is written in detail. In the introduction, please add about the MPOX in immunocompromised patient (in short). If there is any published cases on it, then authors can write it in short in the introduction part.

 Answer: The authors have carefully revised ‘Introduction’ section as per the suggestions of reviewer.

 2. "The first case of Mpox was reported by the United Kingdom Health Security Agency (UKHSA) in the United Kingdom." Please add a reference.

 Answer: The appropriate reference (as given below) is added, as suggested by reviewer.

 Ward T, Christie R, Paton R S, Cumming F, Overton C E. Transmission dynamics of monkeypox in the United Kingdom: contact tracing study BMJ 2022; 379 :e073153 doi:10.1136/bmj-2022-073153

 3. An image of mechanism can be added at Transmission and replication part. (Pathophysiology).

 Answer: The authors have revised the manuscript. However, the pathophysiology is still unknown accurately.

 4. In Ongoing MPXV outbreak 2022-part, one image of world map can be added with the color differentiation of cases.

 Answer: The authors have carefully revised as per the suggestions of reviewer.

 5. In HIV, can we see CD4 count before injecting the vaccine? In that case, in what CD4 count, we can give vaccine? Is there any study on it? If not this paper can recommend this idea for the future. (If authors support my concept of CD4).

 Answer: The authors have carefully revised as per the suggestions of reviewer.

 6. The paper has a good discussion on Mpox vaccine. If authors agree my idea about CD4, can add as a recommendations

 Answers: Whatever scientific literature is available in the databases have been added.

 7. Is it possible to have an info graph on the topic? The Clinicians will get to know the treatment procedure quickly in ward. Like if an immunocompromised patient got admitted with Mpox. How the treatment should be given. What are the drug of choices. PCR is the test of Choice, please add that also in the management and info graph parts.

 Answer: Dear reviewer, Thanks again for your kind suggestion. We described this information in the figure 2.

# Peer review: Reviewer response for version 1

**DOI:** 10.5256/f1000research.143016.r162329

**Published:** 2023-02-13

**Authors:** Osaretin Okonji

**Affiliations:** 1 School of Pharmacy, University of the Western Cape, Cape Town, South Africa

The abstract section looks quite vague. Some more explicit details are necessary

- Please the first 2 sentences in the abstract should be revised and get straight to the point about mpox global outbreak that started in 2022 putting immunocompromised individuals at increased risk.
- Please be consistent- use mpox the new recommended name by the World Health Organization (WHO) in the whole of the manuscript.
- Please remove the symptoms of mpox in the abstract, move to the introduction section or delete. ‘The documented severe signs of monkeypox include widespread rashes with secondary fungal or bacterial skin infections or tissue death (necrosis), intestine obstruction, and difficulties with the heart, lungs, urinary system, and nervous system’.
- It will be good to revise this sentence to: We discussed /reviewed the mpox clinical presentation, available treatment options and current preventive guidelines in immunocompromised patients. ‘The current article goes into great detail about monkeypox disease occurring in immunocompromised patients and preventive guidelines’.

Introduction

- First sentence: please use the new name recommended by WHO, which is mpox
- Please include that WHO has recommended the name mpox to reduce negative stigma linked with the previous name.
- Please include the incubation period of mpox 5–21 days in the first paragraph.
- It will be good to describe the recent outbreak of mpox in 2022 in that first paragraph, but I noticed overlapping of the symptoms of mpox in the abstract and in the other section of the manuscript.
- Please start a new paragraph about the epidemiology of mpox- ‘The epidemiological history of the mpox revealed that…
- In the epidemiology of mpox please include clade 1 and clade 2 strain. 
- The second paragraph in the introduction is not necessary, please delete or move below-
- This first paragraph should also highlight the importance of the review- since there are very few reviews conducted on mpox in immunosuppressed patients.

 The ongoing 2022 mpox outbreak

- First paragraph, the authors should update the viral strain that are now referred to clade 1 and 2 to be consistent with the World Health Organization recommendation. Also include the case fatality rate of 10.6% for clade 1 and 3.6% for clade 2.
- Second paragraph: please include clinical characteristics of the recent mpox outbreak such as HIV infection, sexual transmitted disease

Transmission and replication

- Last paragraph should be moved to the ongoing 2022 outbreak of mpox section and the repetition----- mpox among gays, men to men sex should be taken out.
- Immunosuppression section is not necessary, I suggest it can be briefly written or taken out.

Monkeypox in immunosuppressed patients

- Please update monkeypox to mpox
- Also state detailed information of clinical presentation of mpox among immunosuppressed individuals with regards to the recent outbreak in 2022. This information you can get from CDC website.
- Second paragraph- Please move to prevention and treatment section
- Last paragraph- Please add more studies that have reported the clinical presentations of mpox among immunosuppressed patients, include CDC published findings and other studies from Europe, there are many studies, this is to improve this section because it is an important area of this paper.
**  **

Management and prevention

- The title needs to be revised: Please change to prevention strategies and countermeasures for immunocompromised individuals.
- Please include- creating awareness of the risk factors and educating people about the measures they need to take to reduce exposure to mpox- which is the main prevention strategy for mpox. 
- Please move the fourth paragraph to the first paragraph, it should be placed at the first line after prevention strategy. 
- Third paragraph- ‘In conclusion- please revise
- The fifth paragraph should be moved to the first and second paragraph.

Conclusion and future prospect

- Please indicate further research needed to determine the efficacy of antivirals and also the duration of mpox treatment.
- Please include mpox vaccine efficacy -is necessary to understand the current vaccine efficacy among immunocompromised individuals from the recent vaccination
- Please indicate clinical trials that are currently ongoing for mpox particularly if there is any one for immunocompromised individual’s
- The importance of equitable global distribution of mpox countermeasures and high burden regions/countries should be prioritized.
- Please the conclusion should be succinctly written, and focus should be on the topic.

Is the review written in accessible language?

Yes

Are all factual statements correct and adequately supported by citations?

Yes

Are the conclusions drawn appropriate in the context of the current research literature?

Partly

Is the topic of the review discussed comprehensively in the context of the current literature?

Yes

Reviewer Expertise:

Infectious diseases

I confirm that I have read this submission and believe that I have an appropriate level of expertise to confirm that it is of an acceptable scientific standard, however I have significant reservations, as outlined above.

# Response

**Published:** 2023-03-23

**Authors:** Sirwan Ahmed

**Affiliations:** Rania Pediatric & Maternity Teaching Hospital, Iraq

Authors are very thankful to the reviewer for devoting time for improving the quality of the manuscript. The inputs and suggestions from the reviewer have been taken under consideration and have been implemented in the manuscript.

 The abstract section looks quite vague. Some more explicit details are necessary

 Answer: The authors have revised the abstract and have made it more updated as well as precise, as suggested by the reviewers.

 Please the first 2 sentences in the abstract should be revised and get straight to the point about mpox global outbreak that started in 2022 putting immunocompromised individuals at increased risk.

 Answer: The authors have revised the abstract and have made it more updated as well as precise, as suggested by the reviewers.

 Please be consistent- use mpox the new recommended name by the World Health Organization (WHO) in the whole of the manuscript.

 Answer: The authors have incorporated the suggestion of reviewer throughout the manuscript.

 Please remove the symptoms of mpox in the abstract, move to the introduction section or delete. ‘The documented severe signs of monkeypox include widespread rashes with secondary fungal or bacterial skin infections or tissue death (necrosis), intestine obstruction, and difficulties with the heart, lungs, urinary system, and nervous system’.

 Answer: The authors have revised the abstract, as suggested by the reviewers.

 It will be good to revise this sentence to: We discussed /reviewed the mpox clinical presentation, available treatment options and current preventive guidelines in immunocompromised patients. ‘The current article goes into great detail about monkeypox disease occurring in immunocompromised patients and preventive guidelines’.

 Answer: The authors have revised the abstract, as suggested by the reviewers

 Introduction

 First sentence: please use the new name recommended by WHO, which is mpox

 Answer: The mistake has been rectified by the authors.

 Please include that WHO has recommended the name mpox to reduce negative stigma linked with the previous name.

 Answer: The mistake has been rectified by the authors.

 Please include the incubation period of mpox 5–21 days in the first paragraph.

 Answers: The authors have revised the ‘Introduction’ section

 It will be good to describe the recent outbreak of mpox in 2022 in that first paragraph, but I noticed overlapping of the symptoms of mpox in the abstract and in the other section of the manuscript.

 Answer: The authors have rectified all these mistakes and updated the manuscript with best of the efforts.

 Please start a new paragraph about the epidemiology of mpox- ‘The epidemiological history of the mpox revealed that…

 Answer: The authors have incorporated the suggestion of the reviewer.

 In the epidemiology of mpox please include clade 1 and clade 2 strain.

 Answer: The authors have updated the manuscript as per the suggestion of the reviewer.

 The second paragraph in the introduction is not necessary, please delete or move below-

 Answer: The authors have updated the manuscript as per the suggestion of the reviewer.

 This first paragraph should also highlight the importance of the review- since there are very few reviews conducted on mpox in immunosuppressed patients.

 Answer: The authors have updated the manuscript as per the suggestion of the reviewer.

 Mention about the ongoing 2022 mpox outbreak

 Answer: The authors have updated the manuscript as per the suggestion of the reviewer.

 First paragraph, the authors should update the viral strain that are now referred to clade 1 and 2 to be consistent with the World Health Organization recommendation. Also include the case fatality rate of 10.6% for clade 1 and 3.6% for clade 2.

 Answer: The authors have updated the manuscript as per the suggestion of the reviewer.

 Second paragraph: please include clinical characteristics of the recent mpox outbreak such as HIV infection, sexual transmitted disease

 Answer: The authors have updated the manuscript as per the suggestion of the reviewer.

 Transmission and replication

 Last paragraph should be moved to the ongoing 2022 outbreak of mpox section and the repetition----- mpox among gays, men to men sex should be taken out.

 Answer: The authors have revised the manuscript and rectified the mistakes as per the suggestion of the reviewer.

 Immunosuppression section is not necessary, I suggest it can be briefly written or taken out.

 Monkeypox in immunosuppressed patients

 Answer: Dear reviewer, we believe it is preferable to include this part; kindly accept our suggestion.

 Please update monkeypox to mpox

 Answer: The authors have rectified the mistakes as per the suggestion of the reviewer.

 Also state detailed information of clinical presentation of mpox among immunosuppressed individuals with regards to the recent outbreak in 2022. This information you can get from CDC website.

 Answer: The authors have revised the manuscript as per the suggestion of the reviewer.

 Second paragraph- Please move to prevention and treatment section

 Answer: The authors have revised the manuscript as per the suggestion of the reviewer.

 Last paragraph- Please add more studies that have reported the clinical presentations of mpox among immunosuppressed patients, include CDC published findings and other studies from Europe, there are many studies, this is to improve this section because it is an important area of this paper.

 Answer: The authors have revised the manuscript as per the suggestion of the reviewer.

 Management and prevention

 The title needs to be revised: Please change to prevention strategies and countermeasures for immunocompromised individuals.

 Answer: The authors have revised the manuscript as per the suggestion of the reviewer.

 Please include- creating awareness of the risk factors and educating people about the measures they need to take to reduce exposure to mpox- which is the main prevention strategy for mpox.

 Answer: The authors have revised the manuscript as per the suggestion of the reviewer

 Please move the fourth paragraph to the first paragraph, it should be placed at the first line after prevention strategy.

 Answer: The authors have revised the manuscript as per the suggestion of the reviewer

 Third paragraph- ‘In conclusion- please revise

 Answer: The authors have revised the manuscript as per the suggestion of the reviewer

 The fifth paragraph should be moved to the first and second paragraph.

 Answer: The authors have revised the manuscript as per the suggestion of the reviewer

 Conclusion and future prospect

 Please indicate further research needed to determine the efficacy of antivirals and also the duration of mpox treatment.

 Answer: The authors have revised the manuscript as per the suggestion of the reviewer

 Please include mpox vaccine efficacy -is necessary to understand the current vaccine efficacy among immunocompromised individuals from the recent vaccination

 Please indicate clinical trials that are currently ongoing for mpox particularly if there is any one for immunocompromised individual’s

 Answer: The authors have revised the manuscript as per the suggestion of the reviewer

 The importance of equitable global distribution of mpox countermeasures and high burden regions/countries should be prioritized.

 Answer: The authors have revised the manuscript as per the suggestion of the reviewer

 Please the conclusion should be succinctly written, and focus should be on the topic.

 Answer: The authors have revised the manuscript as per the suggestion of the reviewer

# Peer review: Reviewer response for version 1

**DOI:** 10.5256/f1000research.143016.r162327

**Published:** 2023-02-09

**Authors:** Jawad Basit

**Affiliations:** 1 Department of Medicine, Rawalpindi Medical University, Rawalpindi, Pakistan

This literature review beautifully explains the recently discovered monkeypox infection in the context of immunocompromised patients. The article has given authoritative references for different figures and all the data has been comprehensively put forward and has a natural flow and coherence. The figure is well made and is up to mark. However, I would like to recommend to add more details on the underlying mechanisms of pathophysiological details in immunocompromised individuals. Moreover, the current epidemiological status of monkeypox needs to be more stressed in the introduction ^1^^,^^2^^,^^3^.

Is the review written in accessible language?

Yes

Are all factual statements correct and adequately supported by citations?

Yes

Are the conclusions drawn appropriate in the context of the current research literature?

Yes

Is the topic of the review discussed comprehensively in the context of the current literature?

Yes

Reviewer Expertise:

Cardiology, Novel infections, Malignancies and hematology, gastroenterology

I confirm that I have read this submission and believe that I have an appropriate level of expertise to confirm that it is of an acceptable scientific standard, however I have significant reservations, as outlined above.

## References

[1] : Monkeypox: Potentially another pandemic or a mere hoax?. *Ann Med Surg (Lond)* .2022;81: 10.1016/j.amsu.2022.104364 104364 10.1016/j.amsu.2022.104364 36147163PMC9486631

[2] : A systematic review of the epidemiology of human monkeypox outbreaks and implications for outbreak strategy. *PLoS Negl Trop Dis* .2019;13(10) : 10.1371/journal.pntd.0007791 e0007791 10.1371/journal.pntd.0007791 31618206PMC6816577

[3] : The changing epidemiology of human monkeypox-A potential threat? A systematic review. *PLoS Negl Trop Dis* .2022;16(2) : 10.1371/journal.pntd.0010141 e0010141 10.1371/journal.pntd.0010141 35148313PMC8870502

# Response

**Published:** 2023-03-23

**Authors:** Sirwan Ahmed

**Affiliations:** Rania Pediatric & Maternity Teaching Hospital, Iraq

**Reviewer: 1**

**Comments to the Author**

 This literature review beautifully explains the recently discovered monkeypox infection in the context of immunocompromised patients. The article has given authoritative references for different figures and all the data has been comprehensively put forward and has a natural flow and coherence. The figure is well made and is up to mark.

 Answer: Authors are very thankful to the reviewer for devoting time for improving the quality of the manuscript. The inputs and suggestions from the reviewer have been taken under consideration and have been implemented in the manuscript.

 However, I would like to recommend to add more details on the underlying mechanisms of pathophysiological details in immunocompromised individuals. Moreover, the current epidemiological status of monkeypox needs to be more stressed in the introduction1,2,3.

 Answer: The authors have updated the manuscript using the information gathered from following references

 References

 1. Saeed S, Shabbir H, Basit J, Ur Rehman ME: Monkeypox: Potentially another pandemic or a mere hoax?. Ann Med Surg (Lond). 2022; 81: 104364 PubMed Abstract | Publisher Full Text

 2. Beer EM, Rao VB: A systematic review of the epidemiology of human monkeypox outbreaks and implications for outbreak strategy.PLoS Negl Trop Dis. 2019; 13 (10): e0007791 PubMed Abstract | Publisher Full Text

 3. Bunge EM, Hoet B, Chen L, Lienert F, et al.: The changing epidemiology of human monkeypox-A potential threat? A systematic review.PLoS Negl Trop Dis. 2022; 16 (2): e0010141 PubMed Abstract | Publisher Full Text

 4. Jang W, Kandimalla L, Rajan S, Abreu R, Campos JE. Monkeypox in an immunocompromised patient with underlying human immunodeficiency virus and syphilis infections in Southern Florida of the United States: a case report. AIDS Res Ther. 2023 Feb 18;20(1):12. doi: 10.1186/s12981-023-00504-4. PMID: 36800970; PMCID: PMC9937856.

 5. Dammann F, Raja M, Camargo JF. Progression of human monkeypox infection despite tecovirimat in an immunocompromised adult. Transpl Infect Dis. 2023 Feb;25(1):e14022. doi: 10.1111/tid.14022. Epub 2023 Jan 30. PMID: 36714983

 6. Sharma V, Aggarwal D, Sharma AK, et al. An overview on Monkeypox, Current Paradigms and Advances in its Vaccination, Treatment and Clinical Management: Trends, Scope, Promise and Challenges. *J Pure Appl Microbiol. *2022;16(suppl 1):3000-3012 *. *doi: 10.22207/JPAM.16.SPL1.21

 7. Sharma V, Panwar A, Garg VK, et al. Tecovirimat as a Potential Bioavailable Iinhibitor against MPXVgp158 Established through Molecular Dynamic Simulations and Docking Studies. *J Pure Appl Microbiol*. 2022;16(suppl 1):3168-3178. doi: 10.22207/JPAM.16.SPL1.13

 8. Chandran D, Nandanagopal VG, Gopan M, et al. Major Advances in Monkeypox Vaccine Research and Development – An Update. *J Pure Appl Microbiol. *2022;16(suppl 1):3083-3095. doi: 10.22207/JPAM.16.SPL1.0

 9. Chandran D, Hridya P, Prasanth D, et al. Changing Patterns in the Spread of Human Monkeypox: A Dangerous New Development in Disease Epidemiology. *J Pure Appl Microbiol. *2022;16(suppl 1):3106-3118. doi: 10.22207/JPAM.16.SPL1.11

  10. Thornhill JP, Barkati S, Walmsley S, et al. Monkeypox virus infection in humans across 16 countries: April–June 2022. N Engl J Med. 2022;387(8):679-691. doi:10.1056/NEJMoa2207323

 11. Sigler R, Haidar G. Mpox in immunocompromised patients: with more data, more questions. Transpl Infect Dis. 2023 Feb;25(1):e14023. doi: 10.1111/tid.14023. Epub 2023 Jan 30. PMID: 36714963.

 12. Mitjà O, Ogoina D, Titanji BK, Galvan C, Muyembe JJ, Marks M, Orkin CM. Monkeypox. Lancet. 2023 Jan 7;401(10370):60-74. doi: 10.1016/S0140-6736(22)02075-X. Epub 2022 Nov 17. Erratum in: Lancet. 2022 Dec 3;400(10367):1926. PMID: 36403582; PMCID: PMC9671644.
